# FedKG: A Knowledge Distillation-Based Federated Graph Method for Social Bot Detection

**DOI:** 10.3390/s24113481

**Published:** 2024-05-28

**Authors:** Xiujuan Wang, Kangmiao Chen, Keke Wang, Zhengxiang Wang, Kangfeng Zheng, Jiayue Zhang

**Affiliations:** 1 Faculty of Information Technology, Beijing University of Technology, Beijing 100124, China; xjwang@bjut.edu.cn (X.W.); healer@emails.bjut.edu.cn (K.W.); s202274173@emails.bjut.edu.cn (Z.W.); zhangjiayue@bjut.edu.cn (J.Z.); 2 School of Cyberspace Security, Beijing University of Posts and Telecommunications, Beijing 100876, China; kfzheng@bupt.edu.cn

**Keywords:** social bot detection, federated learning, knowledge distillation, graph neural network

## Abstract

Malicious social bots pose a serious threat to social network security by spreading false information and guiding bad opinions in social networks. The singularity and scarcity of single organization data and the high cost of labeling social bots have given rise to the construction of federated models that combine federated learning with social bot detection. In this paper, we first combine the federated learning framework with the Relational Graph Convolutional Neural Network (RGCN) model to achieve federated social bot detection. A class-level cross entropy loss function is applied in the local model training to mitigate the effects of the class imbalance problem in local data. To address the data heterogeneity issue from multiple participants, we optimize the classical federated learning algorithm by applying knowledge distillation methods. Specifically, we adjust the client-side and server-side models separately: training a global generator to generate pseudo-samples based on the local data distribution knowledge to correct the optimization direction of client-side classification models, and integrating client-side classification models’ knowledge on the server side to guide the training of the global classification model. We conduct extensive experiments on widely used datasets, and the results demonstrate the effectiveness of our approach in social bot detection in heterogeneous data scenarios. Compared to baseline methods, our approach achieves a nearly 3–10% improvement in detection accuracy when the data heterogeneity is larger. Additionally, our method achieves the specified accuracy with minimal communication rounds.

## 1. Introduction

The application of online social networks is becoming increasingly widespread, with the number of users on platforms like Twitter, Facebook, Instagram, and Weibo continuously growing. Social networks have become important tools for users to communicate, entertain, and obtain information. Alongside this, a new type of account controlled by automated programs has emerged on these social platforms [1]: social bots. Social bots can be categorized into malicious and benign accounts. Some benign social bots are designed to automatically aggregate content from various sources and provide services to regular users. However, over time, malicious social bots with nefarious purposes have emerged. These malicious social bot accounts strive to disguise themselves as real human accounts, creating artificial popularity, spreading false information [2], and guiding negative public opinion [3] on social platforms. Evidence of social bots manipulating public opinion for political purposes has been found in events such as Brexit and the 2016 US presidential election [4]. In summary, malicious social bots engage in a range of behaviors that disrupt the social network’s order, posing a significant threat to the social network’s security. Therefore, there is an urgent need for suitable and effective methods for social bot detection to foster a healthy social network environment. In our current research, we examine the differences between social bots and humans, but have not yet provided a detailed classification of social bots.

Existing social bot detection methods focus on the feature information of social accounts, including the user account features, user tweet features, and user relationship features. Graph representation learning methods based on graph neural networks are important approaches for fusing relationship features among users. We notice that most recent social bot detection works are conducted based on isolated data parties. However, utilizing account information from individual data parties for model training has several limitations: (1) the limited number and feature space of social accounts held by a single data party restricts the improvement of model performance [5]; (2) domain-specific bots across different data parties often exhibit similar characteristics [6], and sharing data facilitates the discovery of these common features; (3) labeling social bots is a challenging task, which means a high cost for each data island dedicated to building social bot detection models. Therefore, sharing the data from multiple parties for joint model training can leverage the advantages of a larger user base and richer user feature information, while alleviating the problem of difficult data labeling.

Data privacy is a primary challenge in implementing the data fusion of multi-party account information [7]. Under the premise of complying with relevant regulations and protecting data privacy, data owners often do not share data directly. The key problem lies in how to achieve the joint model training by integrating the multi-party data while ensuring that data privacy is not compromised. Federated learning has emerged as an important method to deal with the related problems.

Additionally, the heterogeneity among multiple data parties, known as the non-Independently and Identically Distributed (non-IID) problem, can significantly impact the performance of federated learning models [8]. For instance, when multiple data owners share their data for training a federated social bot detection model, there are substantial differences in the composition and distribution of social accounts among each participant (e.g., the number of human and social bot accounts present different distributions across multiple platforms). In such cases, utilizing the traditional federated learning methods for direct parameter averaging to obtain a global model can lead to severe biases in the global model, thereby affecting the performance of individual participant models. Consequently, even though federated learning facilitates the integration of multi-party data to some extent, the resulting global model may not positively impact the performance of all participant models. Some existing works [9,10] have improved upon the traditional federated averaging method by applying knowledge distillation. They distill the logit knowledge of client models to the server, resulting in significant improvements compared to existing federated learning algorithms when dealing with data heterogeneity issues among the clients.

Some recent research has focused on federal social bot detection. However, reference [5] fails to acknowledge the significance of the interaction between social accounts in enhancing the detection performance, while reference [6] overlooks the heterogeneity of the data distribution in federated learning. These shortcomings render these methods inadequate for real-world federated social bot detection scenarios. Our approach makes corresponding improvements to address these limitations. In this paper, we combine federated learning with the Relational Graph Convolutional Neural Network (RGCN) [11] graph representation learning method to construct a federated social bot detection model. We apply a class-level cross-entropy loss function to address the class imbalance problem between social bots and human accounts. The knowledge distillation method is applied to optimize and adjust for the data heterogeneity issue among multiple participants. Specifically, our social bot detection model is firstly divided into a feature extraction model based on the graph representation method and a classification model. The method of knowledge distillation is applied to train the global generator and global classification model. The global generator generates pseudo samples with global data knowledge to guide the training of the local classification model, making the decision boundary of the local model closer to the decision boundary of the model trained on global data; the global classification model integrates the logit knowledge of the clients’ local classification models, mitigating the impact of data heterogeneity among clients on model performance. The main contributions of this paper are as follows:

1. We construct a naive Federated RGCN framework for social bot detection in a multi-party data fusion scenario;
2. We define a class-level cross-entropy loss function. and applied it to the training process of local models, mitigating the impact of class imbalance issues during client model training;
3. By applying the knowledge distillation method, we make adjustments at both the server and client sides, effectively alleviating the impact of data distribution heterogeneity among clients on the performance of the federated learning model.

## 2. Related Work

### 2.1. Social Bot Detection

The detection of bot accounts in social media began as early as 2010 [12]. Early social bot detection work primarily involved feature engineering methods, applying the traditional machine learning techniques to classify social accounts. Varol et al. [13] evaluated the classification models using over a thousand features extracted from users’ public data and metadata, including friends, tweets, network patterns, activity time series, and sentiment. Yang et al. [14] achieved an efficient analysis using minimal account metadata and extended it to real-time processing of the entire public tweet stream on Twitter. Kantepe et al. [15] extracted a plethora of features based on the account profiles, tweets, and temporal behavior, and classified accounts using machine learning classifiers.

With the widespread application of deep neural network models, several social bot detection frameworks based on deep neural networks have emerged. Kudugunta et al. [16] detected bots at the tweet level using the content and metadata, employing a deep neural network detection model based on the Long Short-Term Memory (LSTM) architecture. Wei et al. [17] applied a bidirectional LSTM (BiLSTM) network to extract semantic features from tweets. Stanton et al. [18] applied generative adversarial networks for spam detection, relying on limited labeled and unlabeled data to detect spam, thus avoiding labeling costs and inaccuracies. SATAR [19] combines a user’s tweet semantic information, attribute information, and neighbor information for the generalization, simultaneous training on a large number of self-supervised users, and fine-tuning for specific bot detection scenarios to adapt to the evolution of social bots. Hayawi et al. [20] utilized Long Short-Term Memory (LSTM) networks and dense layers to handle a hybrid input model. Arin et al. [21] employed three LSTM models and a fully connected layer to capture the complex behavioral activities of accounts, while exploring three learning schemes to train components connected across different levels.

With the application of graph neural networks for node representation and node classification tasks, many works have emerged that utilize graph models for social bot detection. Alhosseini et al. [22] firstly implemented social bot detection based on graph models by applying the GCN method to obtain the user representation and achieve the classification of the social accounts. BotRGCN [23] applied the relational graph convolutional neural network model to achieve social bot classification, obtaining better detection results. HGT [24] uses relational graph transformers to simulate the heterogeneous influence among users to learn user representations and uses semantic attention networks to aggregate information.

### 2.2. Federated Learning

The continuous development of big data technology has led to the emergence of an increasing number of data silos. Federated learning is an important approach to deal with the data silo problem nowadays. The concept of federated learning was first introduced by McMahan et al. in 2017 [25], with the FedAVG algorithm as the basis. It is a distributed machine learning method that allows data to remain on local clients for model training, eliminating the need for centralized data transmission to a server for centralized learning. The server only needs to aggregate the information learned by the clients to complete the global model update. Currently, federated learning has been widely applied in various fields, such as the medical field [26], the financial field [27], the recommendation field [28], and so on.

The non-IID problem is an important challenge in federated learning that needs to be addressed [29]. In federated learning, multiple participants often have different data characteristics. If a naive federated averaging method is used to simply aggregate the model parameters obtained from the local training of each participant, it often leads to the model falling into a local optimum [30], resulting in an ineffective global model. Our work focuses on the label-based distribution imbalance problem in social bot detection within federated learning, where multiple data contributors have similar data characteristics, but the label distribution of the data are highly imbalanced, leading to inconsistencies between the local optimum and global optima.

Existing optimization methods for the non-IID problem in federated learning can be considered from two perspectives: model-based and data-based. Model-based optimization methods can be further divided into adjustments for server aggregation methods and adjustments for client parameter updates. Adjustments for the server aggregation methods can involve adding weights to the parameters of each client, while adjustments for the client parameter updates include adding regularization terms to the local model updates to reduce the distance between the local and global models, thus addressing the non-IID problem. For example, FedNova [31] improves the model aggregation of FedAvg by normalizing and scaling the local updates of each participant based on their local training epochs before updating the global model. FedProx [32], based on FedAvg, adjusts the client parameter updates by adding a regularization term that constrains the optimization distance between the local and global models. SCAFFOLD [30] uses control variates to correct the client drift in local updates while ensuring faster convergence. FedDyn [33] and MOON [34] constrain the direction of the local model updates by comparing the similarity between model representations, thereby maintaining the consistency between local and global optimization objectives.

The data-based optimization methods can leverage data generation techniques to generate data that makes the data among different participants closer to being identically distributed, thus alleviating the performance loss of the global model caused by data heterogeneity between the clients. FedDistill [35] is a data-free knowledge distillation method, where the participants share the average of label-based logit vectors. FedGAN [36] trains a generative adversarial network (GAN) to handle non-IID data challenges in an efficient communication manner, but inevitably introduces bias. FedGen [37] and FedFTG [38] utilize generator models to simulate the global data distribution for performance improvement.

### 2.3. Federated Social Bot Detection

Most existing works on social bot detection are based on data from a single organization, with few considering the fusion of multi-party data to train powerful models and alleviate the difficulties of labeling social bots for each organization. DA-MRG [6] made the first attempt at federated social bot detection, where the method locally trains a domain-aware multi-relation graph model for social bot detection. Data were divided into multiple participants based on the data size, and the classic FedAvg algorithm was used to aggregate the parameters among the participants to obtain a global detection model. FedACK [5] also focused on the combination of federated learning and social bot detection by extracting features from the user metadata and tweet text for social bot detection. When constructing the federated learning models, it considered the impact of data heterogeneity among multiple participants and proposes a federated adversarial contrastive knowledge distillation method to address the issue of data heterogeneity. These methods do not simultaneously consider an effective social bot detection framework and the heterogeneity of data among multiple participants in federated social bot detection frameworks. To address the aforementioned limitations, we propose a knowledge distillation-based federated graph social bot detection method, achieving more efficient federated social bot detection.

## 3. Materials and Methods

### 3.1. Problem Definition

Given a social network account *U*, we define the basic account information as *P*, tweet information as *T*, and the relationship information as *N*. Our goal is to learn a bot detection function $f:f\left(U\left(P,T,N\right)\right)\to \hat{y}$ that makes the predicted label $\hat{y}$ close to the true label *y*, maximizing the prediction accuracy of the model. For the construction of the federated social bot detection model, we consider the traditional federated learning framework based on the server–client scheme, assuming that there exists a server and *K* clients jointly participating in the training of the federated social bot model. Each client holds a private dataset $D_{k}$, and each client’s dataset $D_{k}$ contains $N_{k}$ individual user information, then the final optimization objective of the problem is defined as:

(1) $$\arg\min_{\theta }L\left(\theta \right)=\frac{1}{K}\sum_{k=1}^{K}\frac{1}{N_{k}}\sum_{i=1}^{N_{k}}\ell \left(x_{i}^{k},y_{i}^{k};\theta \right)$$

*L* is a loss function parameterized by the model parameters θ to evaluate the performance of each client’s prediction model on its private dataset $D_{k}$.

### 3.2. FedKG Social Bot Detection Framework

**Figure 1:**
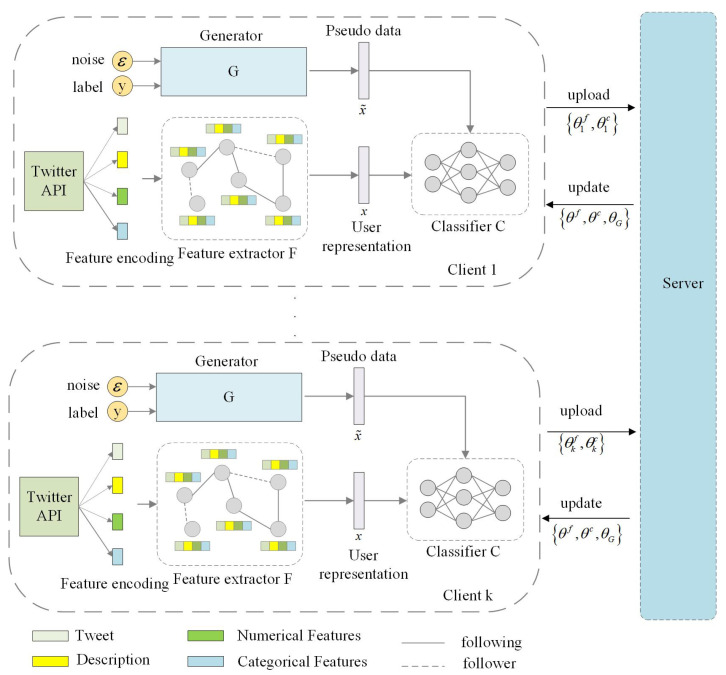
Overview of our proposed FedKG framework.

The overall framework of FedKG is shown in Figure 1. Below, we will introduce the overall model from two components: the local social bot detection framework and the knowledge distillation-optimized federated learning process.

#### 3.2.1. Social Bot Detection Model Based on RGCN Graph Representation Approach

Each client’s local social bot detection model consists of two components: a feature extractor for extracting user feature representations and a classifier for classification, parameterized by $\theta^{f}$ and $\theta^{c}$, respectively, and the feature extractor F and classifier C are trained in stages during the model training process. The details of each component of the local model are specified below.

- **User feature coding**

Firstly, the user tweet information, user description information and user account information are encoded to obtain the initial feature representation of the user. We used a similar encoding approach to BotRGCN [23] to encode user features. For the semantic information in the user’s tweets and descriptions, a pre-trained RoBERTa model [39] is applied for encoding, resulting in representations $r_{t}$ and $r_{d}$; for the user’s numerical features, z-score regularization is applied to obtain the representations $r_{n}$; for the user’s categorical features, one-hot encoding is applied to obtain the representations. These feature representations are then concatenated to form the overall feature representation vector $r_{i}=\left[r_{t},r_{d},r_{n},r_{c}\right]$ of the user, which serves as the initial feature representation of the user.

- **Feature extractor for user feature representation**

The feature extractor is a module that performs the feature extraction on users based on the graph representation learning methods. A social network graph is firstly constructed. In online social networks, the neighbor information of users provides key information for a more accurate analysis of users, resulting in more accurate user feature representations. Therefore, a social network graph is constructed based on the neighbor relationships, and a graph neural network model is applied for feature extraction. Users in the social network are treated as nodes, forming a node set *V*, and the neighbor relationships between users are treated as edges, forming an edge set *E*. There are various types of relationships between users, such as follows, retweeting, and commenting. In this work, the focus is on the mutual following relationships between users. Considering that the following and follower relationships contain different information, the edge set contains two types of edges: one representing the following relationship between users, and the other representing the follower relationship between users. Thus, a heterogeneous graph $G=\left\{V,E\right\}$ is constructed to simulate real-world social networks. We apply the RGCN model as a graph neural network model to learn the low-dimensional feature representations of users. Firstly, we apply a linear layer to transform the initial feature representations of users into the initial representation vectors of nodes in the graph:

(2) $$x_{i}^{\left(0\right)}=\phi \left(W_{1}\cdot r_{i}+b_{1}\right),x_{i}^{\left(0\right)}\in R^{D\times 1}$$

where $W_{1}$ and $b_{1}$ are learnable parameters. After that, L layer R-GCN is applied to learn the user embedding representation vector.

(3) $$x_{i}^{l+1}=\Theta_{\mathrm{self}\;}\cdot x_{i}^{l}+\sum_{r\in R}\sum_{j\in N_{r(i)}}\frac{1}{\left|N_{r}\left(i\right)\right|}\Theta_{r}\cdot x_{j}^{\left(l\right)},x_{j}^{\left(l\right)}\in R^{D\times 1}$$

where Θ is the adjacency matrix, *R* denotes the set of relationship types between users, and $N_{r}$ denotes the set of neighbors of a user with relationship type *r*. After L layers of R-GCN, we obtain the user feature representations $x_{L}$. At this point, the final feature representation of each user is obtained by the feature extractor.

- **Classifier for user classification**

The classifier is used to further transform the user feature representation into the final classification result output. After obtaining the user feature representation through the feature extraction model, we further apply a Multilayer Perceptron (MLP) network to transform the user representation:

(4) $$h_{i}=\phi \left(W_{2}\cdot x_{i}^{\left(L\right)}+b_{2}\right),h_{i}\in R^{D\times 1}$$

where $W_{2}$ and $b_{2}$ are the learnable parameters, and $h_{i}$ is the user feature representation. After that, a softmax layer is applied to transform the transformed user feature representation into a probability distribution:

(5) $${\hat{y}}_{i}=\mathrm{soft}\max\left(W_{O}\cdot h_{i}+b_{O}\right)$$

where $W_{O}$ and $b_{O}$ are learnable parameters.

- **Learning and optimization**

We optimize the feature extractor *F* and classifier *C* with different loss functions during model training. Social bot detection is typically formulated as a binary classification problem. In general, the loss function is defined as the cross-entropy loss function to optimize the model parameters. For example, the loss of the feature extractor and the classifier are defined as follows:

(6) $$\begin{array}{c} L_{ce}^{F}=-\frac{1}{\left|V\right|}\sum_{v_{i}\in V}\left(y_{i}\log{\hat{y}}_{i}+\left(1-y_{i}\right)\log\left(1-{\hat{y}}_{i}\right)\right)\\ L_{ce}^{C}=-\frac{1}{\left|V\right|}\sum_{v_{i}\in V}\left(y_{i}\log{\hat{y}}_{i}+\left(1-y_{i}\right)\log\left(1-{\hat{y}}_{i}\right)\right) \end{array}$$

where $L_{ce}^{F}$ and $L_{ce}^{C}$ are the loss functions of feature extractor *F* and classifier *C*, respectively, *V* is the set of all sample nodes, $y_{i}$ is the true label of the node $v_{i}$, and ${\hat{y}}_{i}$ is the probability that the node is predicted as a positive class by the model.

Considering that, in the social bot detection task, the class imbalance between human and social bot accounts will greatly affect the effectiveness of the training model, the traditional sample-level cross-entropy loss function calculates the loss for each sample and then takes the average. However, if the number of samples in each class is small, their impact on the average loss can be diluted. The optimization algorithm may be biased towards samples from the majority class, resulting in insufficient training of minority class samples. Thus, inspired by DAGAD [40], we modify the traditional sample-level cross-entropy loss function to a class-level cross-entropy loss function to alleviate the issue of local class imbalance for the local model. Specifically, we calculate the loss separately for each class, and then assign the same weight to these losses for summation. In this case, the losses for each class are treated equally, so even if the number of samples in a certain class is small, its loss value still has a greater impact on the model training. The equation is defined as follows:

(7) $$\begin{array}{cc} L_{ce}{}^{\prime }= & -\frac{1}{\left|V_{\min}\right|}\sum_{v_{i}\in V_{\min}}\left(y_{i}\log{\hat{y}}_{i}+\left(1-y_{i}\right)\log\left(1-{\hat{y}}_{i}\right)\right)\\ & -\frac{1}{\left|V_{\mathrm{maj}\;}\right|}\sum_{v_{j}\in V_{\mathrm{maj}\;}}\left(y_{j}\log{\hat{y}}_{j}+\left(1-y_{j}\right)\log\left(1-{\hat{y}}_{j}\right)\right) \end{array}$$

where ${L_{CE}}^{\prime }$ is the class-level cross-entropy loss function, $V_{\min}$ and $V_{\mathrm{maj}}$ represent the sets of minority class sample nodes and majority class sample nodes, respectively, $y_{i}$ is the true label of the node, and ${\hat{y}}_{i}$ is the probability that the node is predicted to be a positive class by the model. Correspondingly, the loss functions of the feature extractor and classifier are modified to class-level cross-entropy loss functions, i.e., $L_{ce}^{F^{\prime }}$ and $L_{ce}^{C^{\prime }}$. The above describes the complete process of the basic social bot detection model for each client.

#### 3.2.2. Knowledge Distillation-Optimized Federated Learning Framework

The overall process of the naive federal learning framework is as follows: the server first initializes the global model parameters θ and distributes the global model parameters to each client; each client trains its local model based on the local private data $D_{k}$, including the training of the local feature extraction model and the local classification model; after the client’s local training is finished, it sends the updated model parameters $\theta_{k}$ to the server for the preliminary average aggregation:

(8) $$\theta_{t+1}=\sum_{k=1}^{K}\frac{N_{k}}{N}\theta_{t+1}^{\left(k\right)}$$

where $\theta_{t+1}$ is the new global model parameters for the next round, *K* is the number of participating clients, $N_{k}$ is the number of samples of client *k*, and *N* is the sum of the number of samples across all clients. This completes one global iteration, and this iteration is repeated many times to obtain the optimal global model.

The data heterogeneity among multiple participants in federated learning has a non-negligible impact on the performance of federated learning. Therefore, in this work, we apply knowledge distillation methods to optimize the federated learning algorithm.

We train a global generator G on the server side, aiming to learn the global data distribution. The parameters of the global generator G are shared among all clients, as shown in Figure 1. Applying standard Gaussian noise z∼N(0,1), given target labels *y*, the global generator generates pseudo-samples $\tilde{x}$ (i.e., user feature representations). These pseudo-samples incorporate data information from all clients. By applying these pseudo-samples to further guide the training of the local classification model, the decision boundary learned by the client model can be closer to the decision boundary of all the global data. This approach mitigates the impact of data heterogeneity among clients and improves the performance of client detection models.

Therefore, the final loss function $L_{k}^{C}$ of the client’s classification model *C* is modified on the basis of the class-level cross-entropy loss function as follows:

(9) $$\begin{array}{cc} L_{k}^{C} & =L_{ce}^{C,k^{\prime }}+L_{dis}^{k}\\ & =L_{ce}^{C,k^{\prime }}+\frac{1}{N_{k}}\sum_{i=1}^{N_{k}}D_{KL}\left(\sigma \left(C_{k}\left(x\right)\right)∥\sigma \left(C_{k}\left(\tilde{x}\right)\right)\right) \end{array}$$

where $L{{}_{ce}^{C,k}}^{{}^{\prime }}$ is the class-level cross-entropy loss of the classifier part calculated by client *k* based on the local real data, $L_{dis}^{k}$ is the difference loss between the probabilities obtained from the real samples and the pseudo samples through the classification model, $N_{k}$ is the number of training samples of the client *k*, $D_{KL}$ is the Kullback–Leibler divergence, $C_{k}$ is the classification model of client *k*, and σ is the softmax function. By minimizing Equation (9), we allow the probability distribution obtained from the real samples approach the probability distribution obtained from the pseudo-samples. This allows the pseudo-samples, which contain knowledge of the global data distribution, to influence the decision boundary of the client’s local classification model.

The global generator *G* is trained on the server using the knowledge distillation method to integrate the data distribution knowledge among global clients. The logits knowledge from the client models serves as the teacher model’s knowledge, while the server’s global generator serves as the student model. Equation (10) is defined to measure the difference in probability distributions between the logit outputs of the client models and the logit outputs of the server model:

(10) $$L_{\mathrm{dis}\;}^{G}=\frac{1}{K}\sum_{k=1}^{K}\sum_{\tilde{x}}\alpha_{t}^{k,y}\left[D_{KL}\left(\sigma \left(C_{k}\left(\tilde{x}\right)\right)∥\sigma \left(C\left(\tilde{x}\right)\right)\right)\right]$$

where *K* is the number of participating clients, $\alpha_{t}^{k,y}$ is the weight of the samples in client *k* with label *y* to the samples in all global data with label *y*, $C_{k}$ is the classification model of client *k*, *C* is the global classification model, and σ is the softmax function. We train the global generator by maximizing Equation (10) to generate pseudo-samples that are beneficial for model training. In addition, a diversity loss is introduced in order to ensure the diversity of samples generated by the generator and to avoid model collapse:

(11) $$L_{\mathrm{var}}=e^{\frac{1}{N^{*}N}\sum_{i,j\in \{1,\ldots N\}}-{∥{\tilde{x}}_{i}-{\tilde{x}}_{j}∥}_{2}^{*}{∥z_{i}-z_{j}∥}_{2}}$$

where ${\tilde{x}}_{i}$ and ${\tilde{x}}_{j}$ are pseudo-samples.

Then, the overall loss of the global generator is:

(12) $$L_{G}=-L_{dis}^{G}+L_{\mathrm{var}}$$

In order to further solve the problem of data heterogeneity among clients, after training the global generator, the server side also applies the knowledge distillation strategy to adjust the global average classifier model, and the loss function of the global classification model training is defined as:

(13) $$L_{C}=\frac{1}{K}\sum_{k=1}^{K}\sum_{\tilde{x}}\alpha_{t}^{k,y}\left[D_{KL}\left(\sigma \left(C_{k}\left(\tilde{x}\right)\right)∥\sigma \left(C\left(\tilde{x}\right)\right)\right)\right]$$

By minimizing Equation (13), the global classification model can further integrate the knowledge from the client classification models, improving the generalization of the global model and enhancing communication efficiency.

## 4. Experiments and Analysis

### 4.1. Experimental Setup

#### 4.1.1. Datasets

We conduct experiments on the Twibot-20 [41] dataset to evaluate our method. The Twibot-20 dataset is a widely used benchmark dataset for social bot detection tasks. It contains a total of 229,573 users. Among them, only 11,826 accounts are labeled, including 5237 human accounts and 6589 bot accounts. It provides the users’ semantic information, account information, and following and follower relationships information among users, supporting the construction of models based on the graph representation learning methods. The user account information used contains numerical features and categorical features, and the specific feature names and feature descriptions are shown in Table 1 and Table 2, respectively.

#### 4.1.2. Baselines

We combine the federated learning framework with the classical RGCN method and apply the knowledge distillation method to optimize the classical federated learning algorithm to address the problem of data heterogeneity among clients. Therefore, the baselines we apply include a detection model trained only locally (named Local), and federated learning methods FedAvg [25], FedProx [32], and FedDistill [35] that address the data heterogeneity problem. We evaluate the performance of these methods under different degrees of data heterogeneity.

- **Local**: A detection model trained only on local private data, without any information exchange between participants;
- **FedAvg**: A basic federated learning algorithm, with parameter sharing between the client and the server, and the server performs weighted averaging of client parameters based on their sample quantities;
- **FedProx**: A federated learning method that introduces a regularization term in the loss function on the clients to reduce the distance between local and global models;
- **FedDistill**: A data-free federated knowledge distillation method where the clients share the average of the label-based logit vectors. Since there is no parameter sharing, the performance of FedDistill decreases significantly. To ensure fairness, we modified the original method to share logit averages based on shared model parameters as a baseline comparison method;
- **FedACK**: A federated social bot detection method that proposes a GAN-based federated adversarial comparison knowledge distillation mechanism. The relationship information between users is not considered in the social bot detection model, and the detection performance has a large gap with the detection of graph model-based methods; we modify its framework by replacing its local social bot detection model with our local detection framework as a baseline comparison method.

#### 4.1.3. Data Heterogeneity

In the experimental validation, we divide the Twibot-20 dataset into multiple parts to simulate multiple participants in federated learning. For data distribution heterogeneity for different participants, we apply the Dirichlet distribution Dir(α) for non-IID partitioning of data to simulate the heterogeneity among different participants in the real world, following a previous work [42], where the parameter α reflects the degree of the data heterogeneity, and a smaller α indicates a higher degree of data heterogeneity among clients.

#### 4.1.4. Implementation Details

The feature extractor utilizes a two-layer RGCN, while the classifier employs a three-layer MLP. The global generator model is an MLP with two linear layers, and the hidden layer dimension is 16. The basic parameters for model training are as follows: batch size is 64, learning rate is 0.01, optimizer is Adam optimizer, and the number of global communication rounds is set to 100.

Regarding the evaluation metrics, our study evaluates the model performance based on the average accuracy of each client model on the test set in each iteration. The experimental results below report the highest average accuracy achieved during the iterations.

### 4.2. Performance Comparison

**Figure 2:**
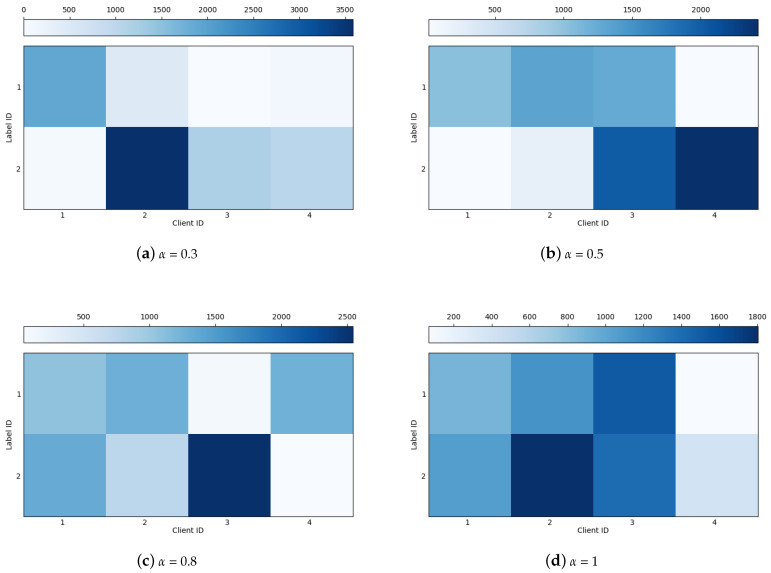
Visualization of data heterogeneity among participants. A darker color means more training samples that a client has for the corresponding label.

We first set the number of clients to four and the number of local training epochs to five. We set the heterogeneity level hyperparameter α to {1, 0.8, 0.5, 0.3} to compare the performance of different methods under different heterogeneity degrees of data distribution. As shown in Figure 2, we visualize the number of human and bot samples at different α, and we can see that as α decreases, the distribution differences in the number of categories among different participants gradually become larger, which represents a major challenge for the construction of traditional federated learning models. Table 3 presents the detection performance of different methods under different degrees of data heterogeneity. It can be observed that our method, FedKG, consistently achieves the highest accuracy at higher degrees of heterogeneity. Specifically, at an α of 0.8, our method has a 3% improvement over the second-highest method, and at an α of 0.5, it has a nearly 10% improvement. Compared to the local training approach, where the clients only utilize their local data for training, the federated learning methods that involve data sharing show significant improvements in performance. At low level of data heterogeneity (α = 1), all of the various federated learning methods achieve relatively high accuracy rates; at higher levels of data heterogeneity, our method demonstrates a clear advantage.

We also conducted an ablation experiment by replacing the class-level cross-entropy loss function with the ordinary sample-level cross-entropy loss, resulting in a variant method called FedKG-C. The experimental results show that after replacing the class-level cross-entropy loss function, the performance of FedKG-C decreases compared to the previous FedKG method under different degrees of data heterogeneity, which indicates that the class-level cross-entropy loss function plays a crucial role in addressing the imbalance issue in social bot detection. Furthermore, it can be observed from the experimental results that the variant method still outperforms other baseline methods when the data heterogeneity is low. This suggests that our method, applying the knowledge distillation method of tuning at both the client and server side, can effectively alleviate the impact of the data heterogeneity problem on the performance of federated learning algorithms, resulting in improved results.

### 4.3. Communication Efficiency Comparison

Table 4 demonstrates the number of rounds required for all algorithms to achieve the specified accuracy under different degrees of data heterogeneity when the number of clients is set to four and the number of local training epochs is set to five. The table shows that under different degrees of heterogeneity, our FedKG method achieves the corresponding accuracy with the minimum number of grounds, showing a clear advantage in terms of efficiency. The reason behind this is that during each ground of FedKG’s federated learning, the knowledge distillation approach is applied. The global generator is trained with the logit information from each client as the teacher knowledge, and pseudo samples with global data distribution are generated to guide the training of the client’s classification model. Additionally, after averaging the server parameters, further training of the global classification model is performed using the client’s classification model logit as knowledge. This method of separate tuning between clients and the server allows for rapid and efficient fusion of knowledge across clients, resulting in a significant improvement in communication efficiency.

**Figure 3:**
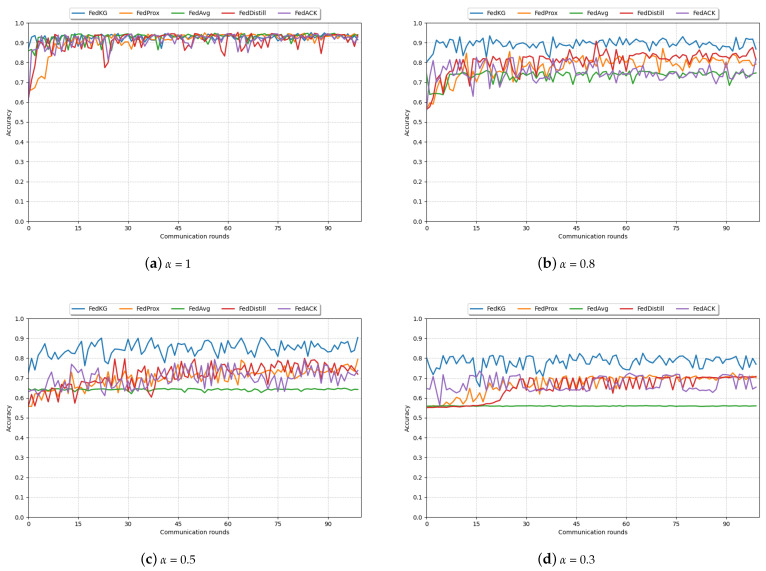
Learning curves of different methods in 100 communication rounds under different α.

Figure 3 shows the model learning curves at 100 global communication rounds for different methods. It can be observed that our method, FedKG, achieves the specified accuracy with the fewest number of communication rounds and maintains relative stability throughout the global iteration process, demonstrating the best performance. The learning curves of both FedProx and FedDistill methods show an increasing and then relatively stable trend. However, FedProx, which addresses the data heterogeneity among clients by adding a regularization term on the client side, requires multiple communication rounds between the server and clients, resulting in poor performance in terms of communication efficiency.

### 4.4. Impact of Local Epochs

**Figure 4:**
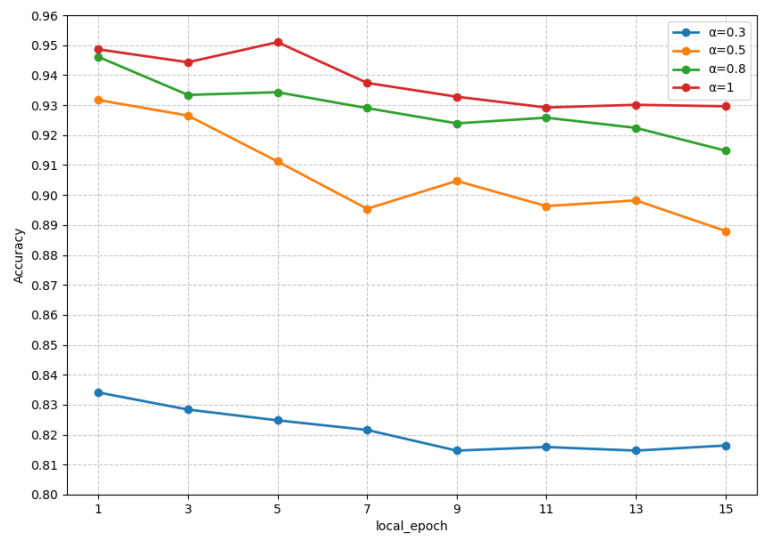
Accuracy of FedKG with different local epochs under different α.

In order to explore the impact of the number of local training epochs on the model’s performance, we conducted experiments on the FedKG method, keeping the number of clients fixed at four and varying the number of local training iterations from 1 to 15. We observed the model performance under data heterogeneity levels, and the experimental results are shown in Figure 4.

From the figure, it can be observed that the model performance generally decreases as the number of local training epochs increases, regardless of the degree of data heterogeneity. This indicates that multiple local training epochs on local clients during the federated model training tend to lead the model towards the local optima which, in turn, results in a decline in the performance of the global model. Therefore, it is crucial to set an appropriate number of local training iterations in federated learning to achieve the best model performance.

### 4.5. Impact of the Number of Clients

We also conducted experiments by varying the number of clients to explore the impact of the number of participants on the model performance in the federated social bot detection problem in cross-organizational scenarios. Table 5 presents the accuracy of the FedKG method under different numbers of clients when the data heterogeneity level α between clients is 0.3, 0.5, 0.8 and 1, respectively. It can be observed that the model achieves the highest accuracy when the number of clients is 2, regardless of the data heterogeneity level. With the increase in the number of clients, the accuracy of the model performance under different degrees of heterogeneity shows a decreasing trend. Moreover, this effect becomes more pronounced when the heterogeneity level is higher. This indicates that, as the number of participants increases, the challenge of data heterogeneity among multiple participants poses a more severe challenge to the performance of federated learning models.

## 5. Discussion

In this section, we discuss some limitations of the present study, further analysis of the experimental results, and future research directions. Firstly, due to the difficulty of obtaining and labeling data in multiple real social platforms, we heterogeneously partition the existing Twibot-20 dataset to simulate multiple data islands in the real world. This partly reflects the effectiveness of our method in training joint models with shared multi-party data. In the future, we will strive to collect data from multiple social platforms to train and verify the model in actual scenarios. Furthermore, in Section 4.5, we discuss the impact of participant numbers on model performance. The results indicate that the model achieves its highest accuracy when the number of clients is two. According to the results presented in Table 5, it is reasonable to infer that with the increase in the number of participants, the data heterogeneity among multiple participants will pose more serious challenges to the performance of the federated learning model. However, this does not necessarily imply that a fixed number of two participants should be employed when developing a federated social bot detection model in real-world scenarios. The determination of the number of participants is also related to factors such as the individual participant data volume, and warrants further comparison and analysis. Finally, we only consider the data heterogeneity issue among different participants and define same model architecture among clients, neglecting the need for different models among participants. In the future, we will pay more attention to the personalized federated learning method and further improve the federated learning algorithm under the premise of model heterogeneity among participants. For example, parameter decoupling can be adopted, where each client retains a portion of the model parameters for the local training without sharing them with the server. This enables each client to learn personalized representations, thereby achieving model heterogeneity across clients.

## 6. Conclusions

The presence of malicious social bots poses a serious threat to social network security. The singularity and scarcity of individual data, coupled with the high cost of labeling social bots, have led to the development of a joint model combining federated learning and social bot detection. We combine the federated learning framework with RGCN model to achieve federated social bot detection. To alleviate the impact of class imbalance in local data, a class-level cross-entropy loss function is applied during local model training. We apply the knowledge distillation method to address the issue of data heterogeneity. Extensive experimental results on benchmark datasets demonstrate the effectiveness of our method in social bot detection, outperforming baseline federated learning methods. In the future, we will further explore the combination of federated knowledge distillation and social bot detection tasks, focusing on the personalization issues in federated learning, and strive to build an efficient framework for federated social bot detection in real scenarios with model heterogeneity among clients.

## Figures and Tables

**Table 1:** Users’ numerical features.

Feature Name	Description
followers	number of followers
followings	number of followings
favorites	number of likes
statuses	number of statuses
active_days	number of active days
screen_name_length	screen name character count

**Table 2:** Users’ categorical features.

Feature Name	Description
protected	protected or not
geo_enabled	enable geo-location or not
verified	verified or not
contributors_enabled	enable contributors or not
is_translator	translator or not
is_translation_enabled	translation or not
profile_background_tile	the background tile
profile_user_background_image	have background image or not
has_extended_profile	have extended profile or not
default_profile	the default profile
default_profile_image	the default profile image

**Table 3:** Comparison of the test accuracy of different methods under different α; a smaller α indicates higher heterogeneity. The bolded value indicates the highest value, and the underlined value indicates the second-highest value.

Setting	α = 1	α = 0.8	α = 0.5	α = 0.3
local	86.09	74.54	64.56	55.96
FedAvg	94.72	75.95	64.75	56.13
FedProx	94.97	87.11	79.37	72.65
FedDistill	**95.25**	90.32	79.88	70.86
FedACK	94.78	82.65	79.27	73.61
FedKG-C	95.03	87.72	81.64	75.76
**FedKG**	95.10	**93.43**	**91.12**	**82.48**

**Table 4:** The round number required for different methods under different α to achieve the specified accuracy.

Setting	α = 1 (acc = 90%)	α = 0.8 (acc = 80%)	α = 0.5 (acc = 70%)	α = 0.3 (acc = 70%)
FedAvg	4	unreached	unreached	unreached
FedProx	13	13	14	34
FedDistill	4	10	24	32
FedACK	5	3	8	3
**FedKG**	2	1	2	1

**Table 5:** Accuracy of FedKG with different number of clients with different α.

Setting	α = 1	α = 0.8	α = 0.5	α = 0.3
2	94.66	94.07	94.08	90.24
4	94.02	93.43	91.12	82.48
6	93.82	92.77	89.94	85.63
8	93.67	88.25	82.99	81.33

## Data Availability

Restrictions apply to the availability of these data. Data were obtained from Shangbin Feng and are available https://github.com/BunsenFeng/TwiBot-20 (accessed on 7 March 2023) with the permission of Shangbin Feng.
